# T-cell Prolymphocytic Leukemia, Cerebriform Variant

**DOI:** 10.7759/cureus.13299

**Published:** 2021-02-12

**Authors:** José Carvalho, Alexandra Esteves, Francisco Teixeira da Silva, Joana Couto, Carlos Ribeiro

**Affiliations:** 1 Internal Medicine, Unidade Local de Saúde do Alto Minho (ULSAM), Viana do Castelo, PRT

**Keywords:** t-cell leukemia, lymphocytosis, adenopathy, splenomegaly, cerebriform

## Abstract

T-cell prolymphocytic leukemia (T-PLL) is a very rare and aggressive lymphoproliferative disorder. We present a 70-year-old man with complaints of fatigue, low urinary output, and peripheral edema for one month. Objectively, he presented diminished respiratory sounds bilaterally and peripheral edema. Analytical study revealed mild anemia and mild lymphomonocytosis, acute kidney injury, and urinalysis with proteins, leukocytes, erythrocytes, and cylinders. Chest radiography was consistent with pleural effusion. Subsequent study showed new onset of thrombocytopenia with a progressive increase of lymphocytosis, in association with inguinal adenopathies and splenomegaly. Immunophenotypic study of peripheral blood and lymph node biopsy were compatible with the diagnosis of T-PLL. Negative serology for human T-cell lymphotropic virus type 1 (HTLV-1) excluded adult T-cell leukemia. Progressive changes in the peripheral blood smear were seen, finally showing the presence of lymphocytes with a cerebriform nucleus, revealing this variant. There was a rapid catastrophic progression, spontaneous tumor lysis syndrome, and death.

## Introduction

Little is known about T-cell prolymphocytic leukemia (T-PLL). It is a rare and aggressive lymphoproliferative disorder composed of post-thymic T cells and usually involves peripheral blood, bone marrow, lymph nodes, and spleen [1,2]. Its incidence is about 0.6/1,000,000 individuals, which corresponds to only 2% of mature lymphocytic leukemias [3]. Of this, only 5% have a cerebriform variant that we found in our case [3].

## Case presentation

We present a 70-year-old man with complaints of fatigue, low urinary output, and peripheral edema progressively increasing for one month. He had no other complaints, namely orthopnea, palpitations, chest pain, cough or sputum, fever, night sweats, or weight loss. He had a medical history of arterial hypertension, dyslipidemia, and Parkinson's disease; he was medicated with losartan, amlodipine, simvastatin plus ezetimibe, rasagiline, levodopa, carbidopa, entacapone, rivastigmine, amantadine, ropinirole, omeprazole, acetylsalicylic acid, mirtazapine, sertraline, diazepam, clonazepam, and quetiapine.

Clinical examination revealed diminished basal respiratory sounds and pitting edema of lower limbs and periorbital edema with anasarca. Laboratory workup (Table 1) showed mild anemia, mild lymphomonocytosis, acute kidney injury, and urinalysis with proteins, leukocytes, erythrocytes, and pathological cylinders.

**Table 1:** Laboratory data on admission

Tests	Reference values	Results
Hemoglobin (g/dL)	13.2 – 17.2	10.7
Leucocytes (/µL)	4.000 – 10.0000	8.920
Neutrophils (/µL)	1.500 – 8.000	3.100
Lymphocytes (/µL)	800 – 4,000	4.300
Monocytes (/µL)	0 – 1.200	1.500
Platelets (/µL)	150.000-400.000	186.000
Urea (mg/dL)	17 – 43	99.8
Creatinine (mg/dL)	0.8 – 1.3	2.36
Sodium (mmol/L)	136 – 145	137
Potassium (mmol/L)	3.5 – 5.1	4.1
Calcium (mg/dL)	8.6 – 10.3	8.7
Phosphorus	2.5 – 4.9	3.7
Lactate Dehydrogenase (UI/L)	125 – 220	379
C-reactive protein (mg/dL)	<0.51	0.40
Sedimentation rate (mm)	2 – 8	17
Brain natriuretic peptide (pg/mL)	<100	97.8
Thyroid-Stimulating Hormone (uUI/mL)	0.35 – 4.94	1.30
Sample urine protein (mg/dL)	negative	70
Urine leukocytes (/field)	0 – 4	5
Urine erythrocytes (/field)	0 – 3	5
Urine pathological cylinders (/field)	0	Some

**Figure 1:**
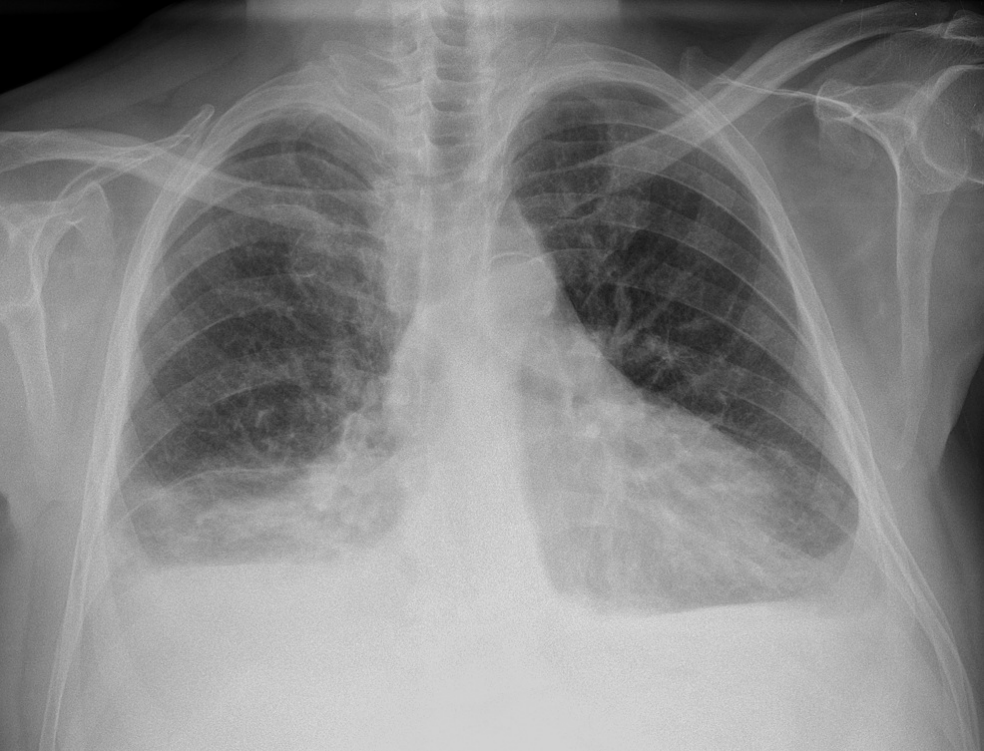
Chest radiograph showing bilateral pleural effusion

Chest radiography was consistent with pleural effusion (Figure 1).

Subsequent studies showed new onset of mild thrombocytopenia and neutropenia, with a progressive increase of lymphocytosis and no improvement of renal function (Table 2).

**Table 2:** Laboratory workup

Tests	Reference values	Day 1	Day 3	Day 5	Day 6	Day 7
Hemoglobin (g/dL)	13.2 – 17.2	10.7	10.2	9.9	10.7	10.4
Leucocytes (/µL)	4.000 – 10.0000	8.920	9.250	8.910	9.010	12.230
Neutrophils (/µL)	1.500 – 8.000	3.100	1.900	2.000	900	400
Lymphocytes (/µL)	800 – 4,000	4.300	5.200	5.500	6.500	9.300
Monocytes (/µL)	0 – 1.200	1.500	1.900	1.200	1.400	1.800
Platelets (/µL)	150.000-400.000	186.000	148.000	124.000	122.000	127.000
Urea (mg/dL)	17 – 43	99.8	103	115	126	139
Creatinine (mg/dL)	0.8 – 1.3	2.36	2.92	3.04	3.11	3.17
Sodium (mmol/L)	136 – 145	137	140	137	137	137
Potassium (mmol/L)	3.5 – 5.1	4.1	3.6	3.3	3.3	3.5
Calcium (mg/dL)	8.6 – 10.3	8.7	8.3	8.0	–	8.0
Phosphorus	2.5 – 4.9	3.7	–	4.2	–	5.0
Uric acid (mg/dL)	3.5 – 7.2	–	9	–	–	10.50
Lactate Dehydrogenase (UI/L)	125 – 220	379	–	423		510
C-reactive protein (mg/dL)	<0.51	0.40	0.91	2.3	2.17	2.1
Beta-2 microglobulin (mg/L)	0.97 – 2.64	–	–	23.4	–	–

**Figure 2:**
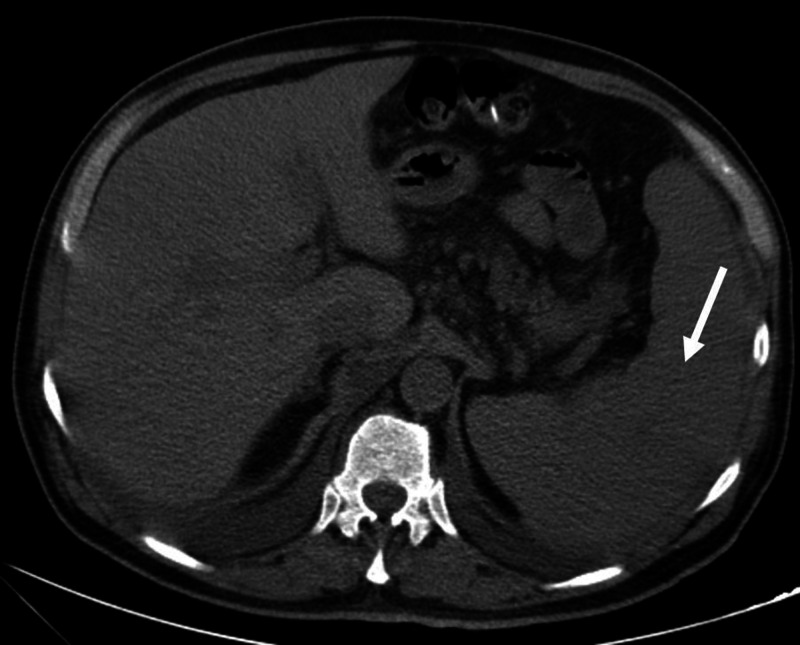
Computed tomography scan An enlarged spleen is seen (white arrow).

Additionally, inguinal adenopathies and splenomegaly became palpable. A computed tomography scan demonstrated a homogeneous 18 cm spleen on day 7 of admission (Figure 2), despite having a normal size spleen reported upon the day of admission. A retrospective review of the admission abdominal ultrasound showed a spleen of 12.8 cm, which was considered within normal limits.

**Figure 3:**
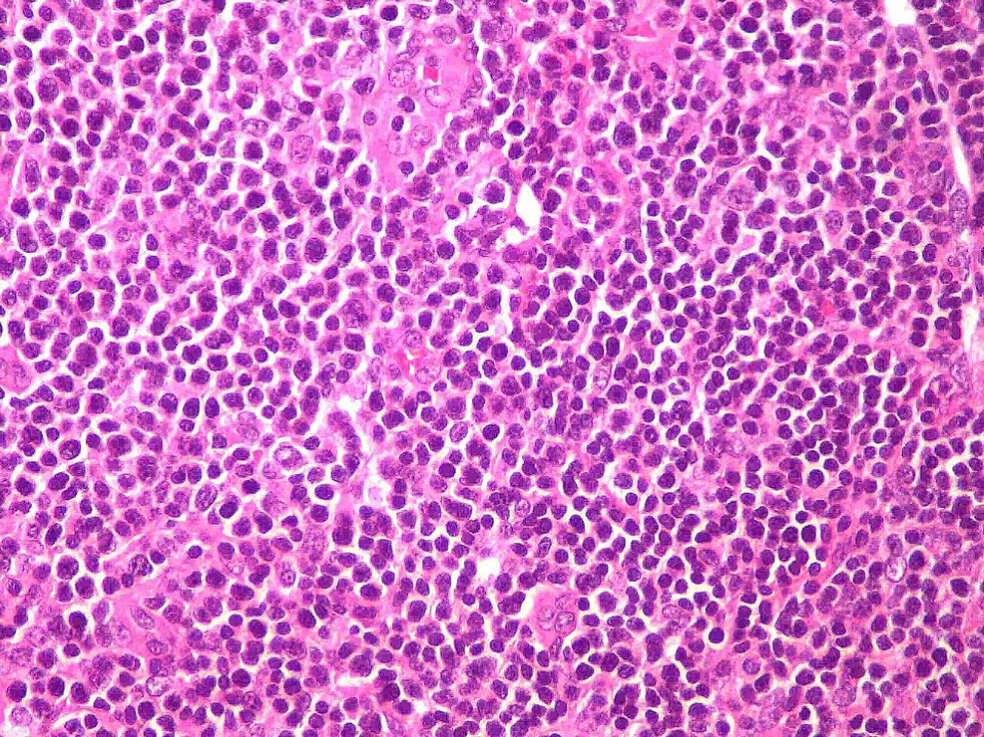
Diffuse proliferation of small T lymphocytes

**Figure 4:**
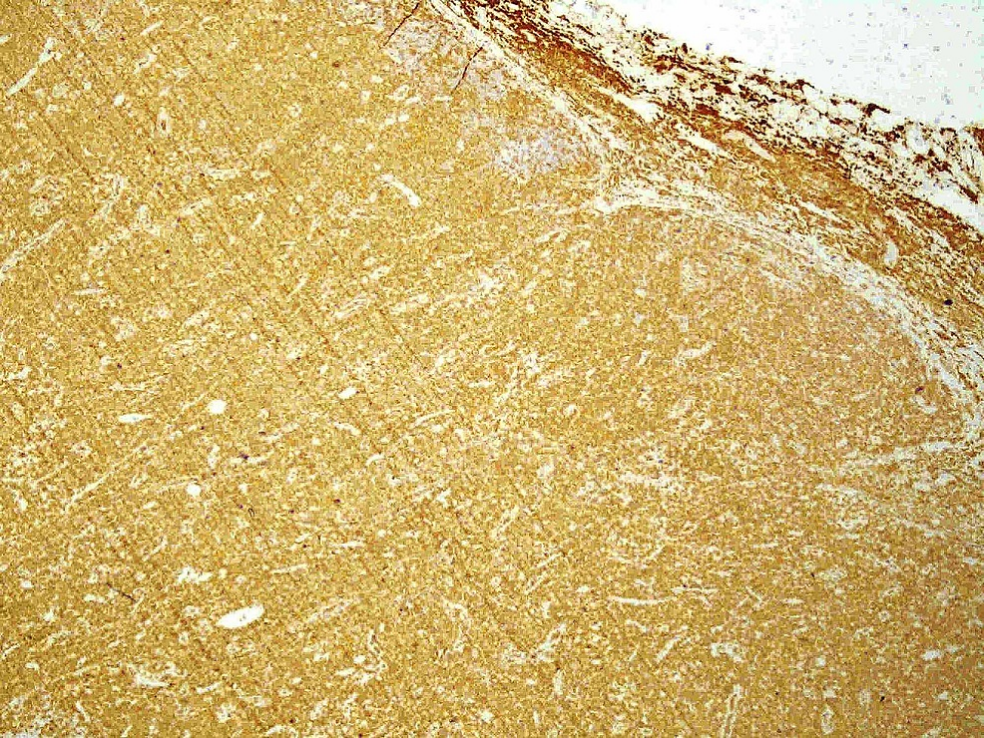
Immunohistochemical study positive for CD3

**Figure 5:**
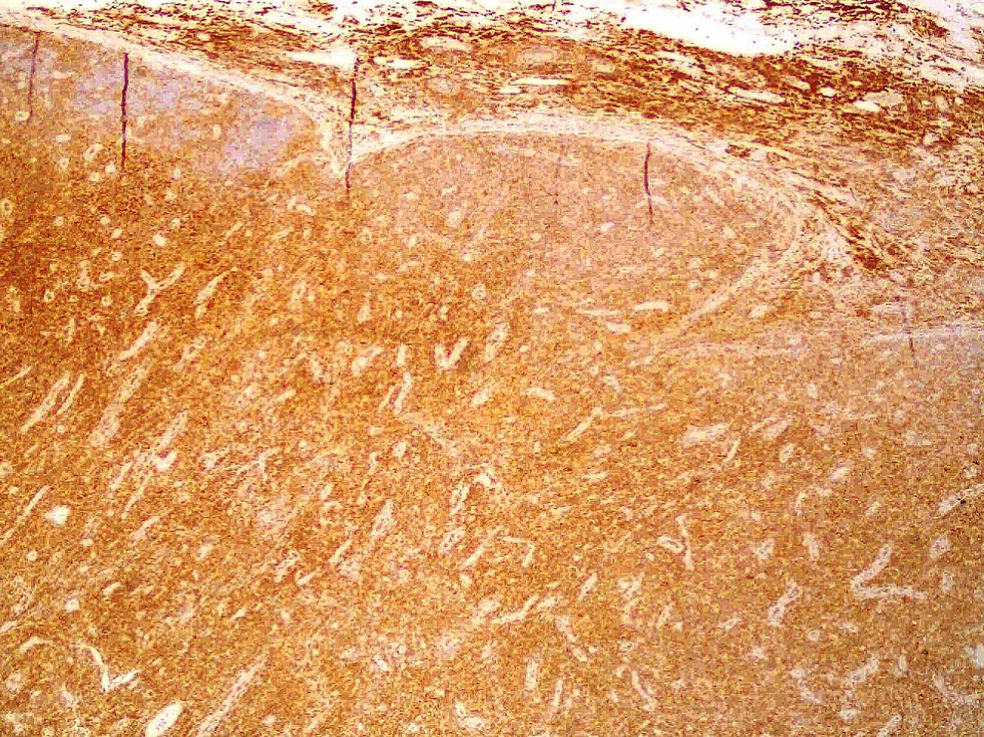
Immunohistochemical study showing marked predominance of CD4 cells

**Figure 6:**
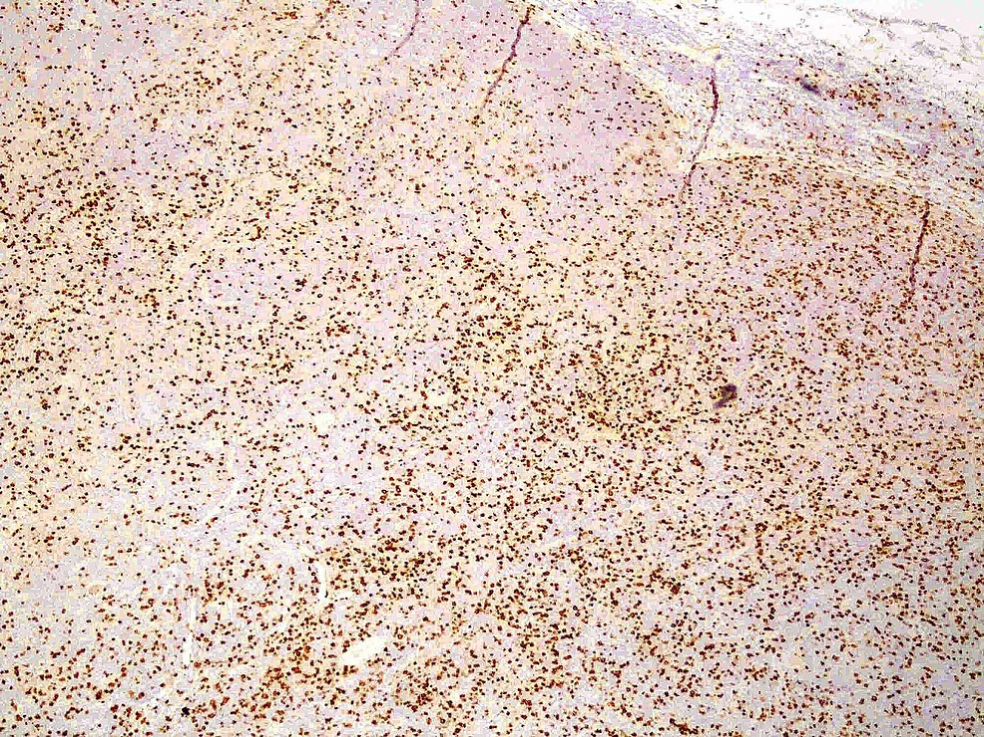
Immunohistochemical study showing fewer CD8 cells

An immunophenotypic study of peripheral blood and inguinal lymph node biopsy was requested. The immunophenotypic study revealed the presence of 9.68x10^3^/µL leukocytes, of which 7.30x10^3^/µL were lymphocytes. Of these, 95.88% were T lymphocytes, of which 94.33% were CD4, 5.53% were CD8, 0.08% were doubly positive, and 0.16% were doubly negative for CD4 and CD8. Of the CD4 cells, 95% were detected to have clonality to the Vbeta2 region of the T-cell receptor (TCR) and to have overexpression of the oncogene TCL1. These findings were compatible with T-PLL. Lymph node biopsy showed diffuse interfollicular proliferation of small lymphocytes with slight atypia (Figure 3) and patent mitotic activity, which in the immunohistochemical study were positive for CD3 (Figure 4), with a marked predominance of CD4 (90% of cells) (Figure 5) over CD8 (only 10% cells) (Figure 6), also compatible with T-PLL. HTLV-1 negative serology excluded adult T-cell leukemia.

The peripheral blood smears revealed progressive changes over day 1 to day 12. Initially, only some lymphocytes with hyperbasophilic cytoplasm were observed, but progressively lymphocytes with a lax chromatin nucleus, abundant lymphocytes with cytoplasmic extensions, and some destroyed leukocytes started to appear. By day 12, cells with irregular nuclei similar to cerebriform nuclei were seen, revealing a cerebriform variant.

Rapidly progressive deterioration of general condition was seen, associated with spontaneous tumor lysis syndrome (acute kidney injury, hyperphosphatemia, hyperuricemia) (Table 2) [4]. Despite the improvement of renal function with intravenous hydration and treatment with rasburicase, the clinical condition still deteriorated, and comfort measures were instituted. The patient died one month after admission.

## Discussion

T-PLL is a rare lymphoproliferative disorder. There is mostly uncertainty about the frequency of various clinical and laboratory findings [5]. This specific case is of particular interest because it presented with mild complaints and with non-specific laboratory changes that quickly escalated with lymphocytosis and acute kidney injury with catastrophic results.

It is interesting to note that no cerebriform cells were seen initially, and only 12 days later did classic cerebriform nuclei predominate.

The lack of consensus on how to diagnose and treat T-PLL led to an international study group creation in May 2017 [3,6]. In 2019, a consensus was reached and published to help standardize diagnosis, treatment, and response evaluation. The aim is to allow the design and conduct of clinical trials in order to improve outcomes in those with T-PLL [3].

There are few clinical trials of therapy of T-PLL. Current research in trial databases, namely clinicaltrials.gov and clinicaltrialsregister.eu, shows three ongoing trials: two in recruiting phase [NCT03989466 and NCT03873493] and one not yet recruiting [NCT04496349]. This highlights the importance that registries may have, helping to capture ongoing empiric data while awaiting formal clinical trial research.

## Conclusions

T-PLL is a clinically aggressive and rare disease, and in such a disease in which there is little certainty as to the frequency of various clinical and laboratory findings, this case is of particular interest because it presented unspecific findings, easily devalued, but which quickly evolved and proved to be catastrophic. Our single case report is a small contribution that adds to the rapid progression of peripheral smear changes that T-PLL can exhibit, including the escalation of cerebriform nuclei variants.
